# A phase I dose-finding and pharmacokinetic study of subcutaneous semisynthetic homoharringtonine (ssHHT) in patients with advanced acute myeloid leukaemia

**DOI:** 10.1038/sj.bjc.6603265

**Published:** 2006-07-18

**Authors:** V Lévy, S Zohar, C Bardin, A Vekhoff, D Chaoui, B Rio, O Legrand, S Sentenac, P Rousselot, E Raffoux, F Chast, S Chevret, J P Marie

**Affiliations:** 1 Inserm CIC 9504, Centre d'Investigations Cliniques, Hôpital Saint Louis, AP-HP, 1 Avenue Claude Vellefaux, Paris 75475, France; 2 Inserm U717, Hôpital Saint Louis, Paris, France; 3 Inserm U717, Département de Biostatistique et Infomatique Médicale, Hôpital Saint Louis, AP-HP, Paris, France; 4 Service de Pharmacie Pharmacologie Toxicologie, Hôtel Dieu de Paris, AP-HP, Paris, France; 5 Département d'Hématologie et d'Oncologie Médicale, Hôtel Dieu, AP-HP, Paris, France; 6 Service d'Hématologie Clinique, Hôpital Saint Louis, AP-HP, Paris France

**Keywords:** homoharringtonine, phase I, continual reassessment method, stopping rules, leukaemia

## Abstract

To determine the maximum-tolerated dose (MTD), dose-limiting toxicities and pharmacokinetic of semisynthetic homoharringtonine (ssHHT), given as a twice daily subcutaneous (s.c.) injections for 9 days, in patients with advanced acute leukaemia, 18 patients with advanced acute myeloid leukaemia were included in this sequential Bayesian phase I dose-finding trial. A starting dose of 0.5 mg m^−2^ day^−1^ was explored with subsequent dose escalations of 1, 3, 5 and 6 mg m^−2^ day^−1^. Myelosuppression was constant. The MTD was estimated as the dose level of 5 mg m^−2^ day^−1^ for 9 consecutive days by s.c. route. Dose-limiting toxicities were hyperglycaemia with hyperosmolar coma at 3 mg m^−2^, and (i) one anasarque and haematemesis, (ii) one life-threatening pulmonary aspergillosis, (iii) one skin rash and (iv) one scalp pain at dose level of 5 mg m^−2^ day^−1^. The mean half-life of ssHHT was 11.01±3.4 h, the volume of distribution at steady state was 2±1.4 l kg^−1^ and the plasma clearance was 11.6±10.4 l h^−1^. Eleven of the 12 patients with circulating leukaemic cells had blood blast clearance, two achieved complete remission and one with blast crisis of CMML returned in chronic phase. The recommended daily dose of ssHHT on the 9-day schedule is 5 mg m^−2^ day^−1^.

Homoharringtonine (HHT) (Cephalotaxine, 4′-methyl (2′*R*)-hydroxy-2′-(4″-hydroxy-4″-methylpentyl)-butanedioate (ester), [3(R)]- (9CI)) is a cephalotaxus alkaloid obtained by extraction from the evergreen tree *Cephalotaxus* sp present in China. Seeds of this genus of conifers were part of the traditional Chinese medicine (Huang *et al*, 1983). In the 1970s, collaboration between US and Chinese investigators led to isolation of the active extract of *Cephalotaxus.* Homoharringtonines (and its analogues) are inhibitor of protein synthesis with an effect on DNA that may be important. Their effects are dose and time dependent (Huang, 1975). Homoharringtonine was found to be a selective inhibitor of transpeptidation during the elongation cycle (Fresno *et al*, 1977; Tujebajeva *et al*, 1989; Zhou *et al*, 1995). Moreover, recent studies on chronic myeloid leukaemia (CML) suggest both apoptosis and differentiation as potential downstream effectors of HHT (Kuliczkowski, 1989; O'Brien *et al*, 1993; Visani *et al*, 1997). Homoharringtonine had been shown to induce apoptosis in different types of leukaemic cells *in vitro* and the HHT-induced apoptotic cascade is characterised by cytochrome *c* release and caspase activation (Cai *et al*, 2001). More recently, HHT has been shown to regulate vascular endothelial growth factor expression in K562 cells (Ye and Lin, 2004). Preclinical studies demonstrated a wide range of cytotoxic effects mainly directed at rapidly proliferating cells, irrespective of their lymphoid or myeloid origin (Takemura *et al*, 1985; Baguley *et al*, 1989). *In vitro* combination with other cytotoxic agents was consistently nonsynergistic with the notable exception of cytarabine (Wilkoff *et al*, 1989; Zhou *et al*, 1990).

Original studies of HHT in cancer and leukaemia patients were dated from the 1970s in China. Phase I trials were later performed in the US using purified alkaloids. Chinese studies were summarised by Grem *et al* (1988) with data available for 350 patients with leukaemia. Almost 25% of the acute myeloid leukaemia (AML) patients achieved complete remission (CR), but these results were difficult to evaluate (Grem *et al*, 1988). In US, purified HHT was tested first in bolus infusion (Neidhart *et al*, 1983; Legha *et al*, 1984; Stewart and Krakoff, 1985) and later replaced by continuous infusion schedule to avert HHT-associated cardiovascular complications (Coonley *et al*, 1983; Neidhart *et al*, 1986). Five phase I/II clinical studies in patients with acute leukaemia were conducted (excluding myelodysplasia (MDS), APL or paediatric AML) with variable schedule of continuous infusion with CR rate ranging from 0 to 25% (Warrell *et al*, 1985; Kantarjian *et al*, 1989; Feldman *et al*, 1992a, 1992b) (for complete reference, see Kantarjian *et al* (2001)).

Homoharringtonine is currently widely used in China for the treatment of AML, but the extraction from the barks of *Cephalotaxus* sp raised the problem of extinction of the raw material (scarcity and poor growth rate of *Cephalotaxus* sp) and the presence of impurities (at least 1% of related compounds). Therefore, a highly purified semisynthetic HHT, using the leaves of the tree, was produced by Oncopharm Corporation with the perspective of registration.

In this context, we conducted a sequential phase I dose-finding clinical study in order to determine the maximum-tolerated dose (MTD) and pharmacokinetic of semisynthetic homoharringtonine (ssHHT). We choose the subcutaneous (s.c.) route to facilitate the administration of the drug, especially in the elderly patients, given as a twice daily s.c. injection for 9 days, in patients with advanced myeloid leukaemia.

## PATIENTS AND METHODS

### Eligibility (patients' selection)

The study was approved by the Local Ethics Committee at the Hôtel Dieu Hospital. Adult patients (>18 and <80 years old) with a WHO performance status ⩽3 and with documented advanced myeloid malignancies were eligible for the study, that is, either (i) AML refractory to one (age >60 years) or two (⩽60 years) induction courses of chemotherapy or in first relapse or more; relapse after autologous stem cell transplantation or allogeneic stem cell transplantation without possibility of allogeneic lymphocytes re-injection; (ii) accelerated or acute myeloproliferative disorders not responding to conventional treatments; (iii) MDS refractory to chemotherapy or in relapse after treatment (chemotherapy or stem cell transplantation); (iv) CML in accelerated or acute phase or refractory to interferon *α*. Patients provided signed, informed consent. Those patients who received chemotherapy within 4 weeks of study entry, with active severe infection, and with cardiac, renal or liver dysfunctions, were excluded. Pregnant and lactating women were excluded.

### Treatment plan and follow-up studies

On entry, all patients received appropriate supportive care, including hydratation, alkalinisation of the urine, allopurinol at the discretion of the treating physician and diagnostic evaluation and treatment of the documented or suspected infections. Prophylactic antiemetics were given to all patients. Menstruating women received hormone therapy to prevent menstruation. Patients were transfused to maintain haemoglobin level >8 g dl^−1^ and platelets >10 × 10^3^ *μ*l^−1^, or as clinically indicated. Haematopoietic growth factors were not permitted, with exceptions granted at the discretion of the principal investigator.

Protocol treatment consisted of one 9-day course of chemotherapy with ssHHT administered by s.c. route twice a day. Following the cycle of chemotherapy, an additional course of ssHHT could be administered in case of response at the discretion of the treating physician, or any other additional chemotherapy. Semisynthetic homoharringtonine was manufactured by Oncopharm (Le Mans, France).

### Trial design and dose allocation rule

The design of this dose-finding phase I clinical trial was chosen to assess the MTD of ssHHT in the treatment of patients with advanced myeloid leukaemia. The MTD was defined as the dose that achieves a dose-limiting toxicity (DLT) in 33% of patients. Five dose levels were tested, namely 0.5, 1, 3, 5 and 6 mg m^−2^ day^−1^. The continual reassessment method (CRM) (O'Quigley *et al*, 1990; Garrett-Mayer, 2006; O'Quigley and Zohar, 2006) was used as the dose allocation rule in the trial. It is based on a mathematical modelling of dose–DLT relationship, iteratively updated using Bayes theorem along the trial, as follows. First, before trial onset, each dose level is arbitrarily associated with initial guesses of DLT probability by the investigator. These initial guesses, which relied on his (her) personal experience and on literature, were fixed at 0.05, 0.1, 0.15, 0.33 and 0.5, respectively. The uncertainty in this dose–DLT relationship is incorporated into a prior. Then, patient accrual begins, with grouped inclusions of three patients per dose level. The first cohort is administered the first dose level. Then, on the basis of observed responses (DLT or not) of patient cohort, DLT probabilities of all dose levels are updated using Bayes theorem. The dose level associated with an updated DLT probability close to 33% is recommended to be administered to the next patient cohort. All this process is re-run until fixed sample size is reached, or in case of fulfilled stopping criteria measuring futility of trial continuation (Zohar and Chevret, 2001).

### Assessment of toxicity and response

Dose-limiting toxicity was defined, using National Cancer Institute Common Toxicity Criteria (version 2.0), as at least grade 2 neurological or urinary toxicities, at least grade 3 cardiac toxicity and higher than grade 3 pulmonary and digestive toxicities. All other toxicities were considered as limiting if they were graded at least 3. Persistent myelosuppression defined by neutrophil <500 *μ*l^−1^ and/or platelets <20 000 *μ*l^−1^ associated with empty bone marrow for 40 days or more was also considered as DLT. Local tolerance at the point of injection was analysed apart from pain and local reaction.

Patient response was evaluated by the examination of marrow and peripheral blood smears, and assessed using criteria established by the National Cancer Institute workshop (Cheson *et al*, 1990). Complete remission was defined as follows: absolute neutrophil count ≥1500 *μ*l^−1^, platelets ≥100 × 10^3^ *μ*l^−1^, absence of leukaemic blast in peripheral blood, >20% cellularity of bone marrow with maturation of all the cell lines, <5% leukaemic blasts in the bone marrow and absence of Auer rods. Partial remission was similarly defined, except that the percentage of blast in the marrow was between 5 and 20%, and Auer rods could be present as long as the percentage of the blasts was ⩽5%. Bone marrow leukemic cell clearance was checked at the end of ssHHT treatment (D10) if no leukaemic cells were circulating. If there was a response on the basis of peripheral blood differential, bone marrow examination was performed at day 28 or whenever the ANC recovered to more than 1 × 10^3^ *μ*l^−1^, and in any case at day 40 in case of persisting pancytopaenia without circulating leukaemic cells.

### Pharmacokinetic studies

Blood samples (6 ml) were collected in sodium heparin tubes at 12 h after the administration on day 3, at 0.25, 0.5, 0.75, 1, 2, 4, 8 and 12 h after the administration on day 5 and at 4, 8, 12, 24, 36 and 48 h after the last injection on day 9. Samples were immediately centrifuged at 3000 **g** for 10 min and then plasma was divided into two aliquots of at least 1.5 ml and frozen at −20°C until the time of analysis. Plasma samples were assayed by a specific and sensitive liquid chromatographic assay with fluorimetric detection by monitoring the emission at 320 nm with excitation wavelength of 280 nm. Analytical column was a 5-*μ*m Lichrospher® C18 column (250 mm × 4 mm I.D.) (Merck, Darmstadt, Germany). Sample preparation (1 ml) consisted of addition of internal standard (0.1 ml of quinidine 50 *μ*g ml^−1^), solvent extraction 2.5 ml of ethyl acetate and evaporation to dryness for 50 min at 60°C. The residue was dissolved in 100 *μ*l of mobile phase. An 80 *μ*l aliquot was injected onto the column. The mobile phase was tetrahydrofurane-acetonitrile-citrate/phosphate buffer adjusted to pH 4.2 (5/15/80, v v^−1^). Flow rate was 0.8 ml min^−1^. The retention times of ssHHT and IS quinidine were 5.4 and 8.4 min, respectively. Between-run and within-run coefficients of variation, measured at nine concentrations (1–60 ng ml^−1^), were less than 11.0%. The lower limit of quantification of this assay was 1 ng ml^−1^.

Estimates of pharmacokinetics parameters for ssHHT were derived from individual concentration–time data sets by noncompartmental analysis using the software package WinNonLin version 4.0 (Pharsight Corporation, Mountain View, CA, USA). The peak plasma, trough plasma concentrations and the time to peak concentrations were the observed values observed at day 5. The area under the plasma concentration *vs* time curve (AUC) was calculated using the linear trapezoidal method from time zero to the time of the final quantifiable concentration. The AUC was then extrapolated to infinity (AUC_inf_) after the last injection by dividing the last measured concentration by the rate constant of the terminal phase (*k*), which was determined by linear regression analysis of the final three or four time points of the log-linear concentration–time plot. The apparent s.c. total plasma clearance of ssHHT (CL/F) was calculated by dividing the administered dose by the observed AUC_inf_. The apparent volume of distribution (*V*_*d*_*β*/*F*) was defined as CL/*k* after the last injection. The terminal elimination half-life (*T*_1/2_) was defined as ln 2/*k* after the last injection at day 9. Pharmacokinetic data were reported as mean and standard deviation.

### Statistical estimation

All along the trial, updated DLT probability of each dose level was iteratively computed after each completed cohort of three patients, using the BPCT software (Zohar *et al*, 2003). Stopping criteria, aiming at detecting futility of trial continuation, were also computed. They are based on the computation of predictive gains from further inclusions, both on the estimated DLT probability of the MTD and on precision of DLT probability of the MTD as measured on the width of its 95% credibility interval. Trial is recommended to stop when expected gains appear too tiny (ref.).

Qualitative and quantitative results were expressed as percentage and median (Q1–Q3), respectively, using R 8.1 software (the R development core team, 2003). Survival curve was estimated by the Kaplan–Meier method, using SPLUS2000 software (MathSoft Inc., Seattle, WA, USA). The terminal elimination half-life of ssHHT was analysed as a function of ssHHT dose level using the Kruskal–Wallis test.

## RESULTS

### General

Between February 2001 and July 2003, 19 patients were registered into the study. The median age of study participants was 57 years (range, 20–79 years), with eight patients (42%) aged over 60 years. There were eight men (42%) and 11 women (58%). Three (16%) subjects had a baseline performance status (WHO) of 0, 8 (42%) of 1, 7 (37%) of 2 and (5%) of 3. Sixteen (84%) patients had an AML, among them two (10.5%) had secondary AML (one MDS and one CMML). Two (10.5%) patients had accelerated CML and one acute phase of CMML (5%). Among the 16 AML patients, seven (44%) were in first relapse, three (19%) in second relapse and three (19%) in third relapse. One (6%) patient was in first relapse after allogeneic stem cell transplantation and two (12.5%) patients were primary refractory to chemotherapy. Among the cohort, nine patients (47%) had received high-dose Ara-C, two (10%) were allografted and two (10%) were autografted.

Among the 19 patients, 18 (95%) were treated and evaluable. One patient (5%) died from progressive disease before evaluation. Thus, all the results are presented on the 18 evaluated patients.

### Estimation of the MTD

The assigned ssHHT dose levels and observed responses in terms of DLT of the 18 assessable patients are listed in Table 1. The first cohort of three patients was treated at 0.5 mg m^−2^ day^−1^ with no DLT. None of the three patients exhibited DLT, resulting in updated DLT probabilities for the five dose levels of 0.001, 0.003, 0.006, 0.035 and 0.11, respectively. As the fifth dose level was associated with DLT probability of 0.11, close to 0.33, it should have been recommended to the next three patient cohorts. Nevertheless, for ethical reasons, investigators preferred not to skip up to the fifth highest dose level, but the third dose level. Thus, the second patient cohort was administered 3 mg m^−2^ day^−1^. One DLT out of three was observed. Incorporated in the analysis, the updated dose–DLT relationship showed increased DLT probability at each dose level. The fourth dose level, 5 mg m^−2^ day^−1^, was then recommended from the third up to the sixth patient cohort.

Based on the six patient cohorts, stopping trial decision was made by the expert committee, with three over four stopping criteria detecting futility of trial continuation. The estimated DLT probability associated with the dose level of 5 mg m^−2^ would have been expected to change by less than 5% even if three further patients were included.

At the end of the trial, the MTD was selected to be the fifth dose level, that is, 5 mg m^−2^ day^−1^, with estimated DLT probability of 36.1% (95% credibility interval: 15.8–58.6%). Dose-limiting toxicities were recorded in four (33%) out of the 12 patients who actually received the 5 mg m^−2^ day^−1^ ssHHT-loading dose.

### Toxicity

The designation and grade of all significant toxicities observed during the treatment are summarised in Table 2a. Five DLTs were observed, one grade 3 hyperglycaemia with hyperosmolar coma at 3 mg m^−2^ requiring insulin and rehydration, and four DLTs at dose level of 5 mg m^−2^ day^−1^: (i) one anasarque and haematemesis, (ii) one life-threatening pulmonary aspergillosis, (iii) one skin rash and (iv) one scalp pain. The only significant toxicities experienced by the three patients who received the starting dose of 0.5 mg m^−2^ day^−1^ were grade 2 digestive toxicities in two patients and grade 2 infectious disease in one patient. The second patient entered into the first dose level died at day 19 owing to progressive disease (AML). Similarly, three patients were treated with the third dose level (3 mg m^−2^ day^−1^) without any notable drug-related toxicities, aside from one grade 3 endocrine (diabetes with hyperosmolar coma) and one grade 3 infectious episode (systemic candidosis) observed in the same patient. Twelve patients were treated at the estimated MTD (5 mg m^−2^ day^−1^); in this setting, five grade 3 and one grade 4 toxicities were noted. Grade 3 toxicities were one infectious (pneumopathy), one digestive (haematemesis), one cardiovascular (anasarque), one pain (scalp) and two cutaneous. Grade 4 toxicity was an invasive aspergillosis (Table 2b).

Myelosuppression occurred in 100% of the patients, with a median duration of 31 days (24–45 days).

Local tolerance was carefully monitored for all patients. Six (33%) patients had a grade 1 pain at the site of injection, 12 patients (67%) experienced local erythema and three (17%) a local haematoma. From the 313 injections given, 16 (5%), 70 (22%) and 6 (2%) presented pain, local erythema or haematoma, respectively. All theses toxicities were grade 1.

### Response and survival

**Figure 1:**
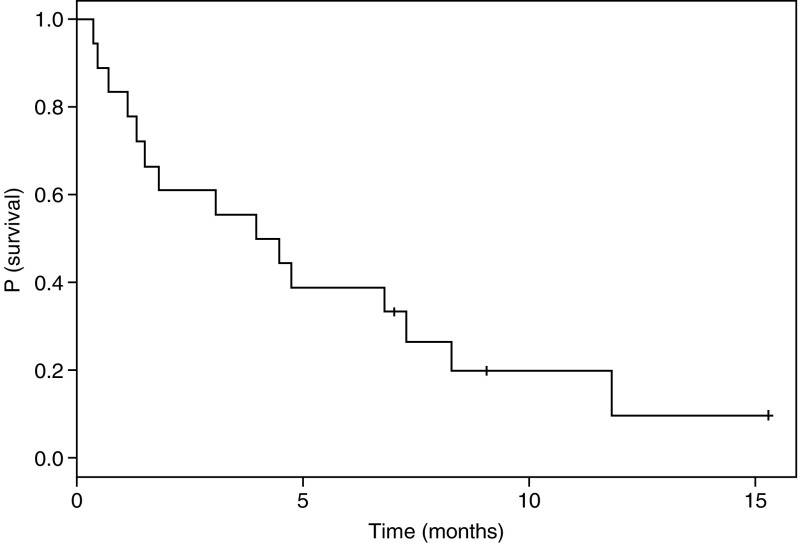
Kaplan–Meier survival curve in treated patients (*n*=18).

Antileukaemic effect is summarised in Table 3. Blood leukaemic clearance occurred in 12 patients with leukaemic-circulating cells, including one patient at 0.5 mg m^−2^ and two at 3 mg m^−2^. Bone marrow blast clearance at the end of chemotherapy was evaluated at day 10 in 14 patients. Five patients showed an empty bone marrow, one out of three patients at 3 mg m^−2^ and four out of 10 patients at the DLT. Two patients (11%) achieved CR (duration 4 months and 3 months, respectively), and one patient with blast crisis of CMML return in chronic phase for 6 months. These three patients had been treated at the estimated MTD (5 mg m^−2^ day^−1^). The two patients who achieved CR had been treated for AML in first relapse and second relapse, treated by conventional daunorubicine Ara-C combination and high-dose Ara-C, respectively. The patient with CMML who returned to chronic phase was treated after the failure of daunorubicin and conventional doses of Ara-C. The median survival of the 18 treated patients was 4.2 months and survival at 6 months was 38.9% (95% CI, 21.8–69.4%) (Figure 1).

### Pharmacokinetics

**Figure 2:**
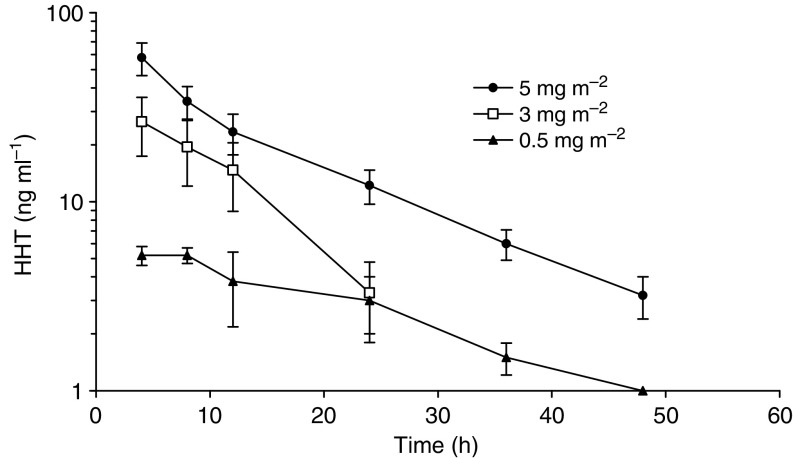
Concentration–time profiles of HHT at dose levels ranging from 0.5 to 5 mg m^−2^ day^−1^ after last injection at day 9. Data from the same dose levels were grouped and are presented as mean values (symbols)±s.e. (error bar). The legend indicates each of the dose levels used.

Pharmacokinetic studies were performed in 17 patients with complete concentration–time profiles available. Figure 2 shows plasma concentration *vs* time profiles of ssHHT at day 9 after the last injection at the dose levels of 0.5 mg m^−2^ day^−1^ (*n*=3), 3 mg m^−2^ day^−1^ (*n*=2) and 5 mg m^−2^ day^−1^ (*n*=12). The mean pharmacokinetic parameters of ssHHT are summarised in Table 4.

Interpatient variability in pharmacokinetic parameters was low, more particularly for *C*_max_ at the 5 mg m^−2^ day^−1^ level (CV=21.1%). *T*_max_ was short and consistent with s.c. absorption (range 0.25–1.1 h). The AUCs but not *C*_max_ increased in near proportion with increasing doses of ssHHT. Disappearance of ssHHT from the central plasma compartment was characterised by elimination in an apparent monoexponential manner. The estimate apparent terminal half-life was relatively consistent in all patients, exhibiting a mean value of 11.01±3.4 h when all doses were combined. *T*_1/2_ was dose independent (*P*=0.52). Evolution in *C*_min_ between days 5 and 9 shows no apparent accumulation of ssHHT. Plasma concentrations of ssHHT were still detectable 48 h after last injection at day 9.

## DISCUSSION

Traditional induction regimen for AML, such as combination of cytarabine and an anthracycline, consistently leads to CR; however, relapse is common. Only 25–45% of patients younger than 60 years remain in CR at 4 years (Bennett *et al*, 1997). Patients who develop AML after a pre-existing MDS have even worse responses (Kantarjian *et al*, 1997). New approaches are therefore needed to address this problem.

Homoharringtonine has demonstrated promising activity in several haematological malignancies, and has been extensively studied in the 1980s, demonstrating an interesting efficacy in CML and AML. However, hypotension was the DLT when HHT was used at high doses and/or in short schedule infusions, whereas myelosuppression was DLT for continuous low-dose infusions. Nonhaematologic toxicities were considered as mild and reversible. Moreover, despite its significant antileukaemic activity, its difficult production, the unreliability of source supply, the difficulty of extraction and the low level of purity of product limited its evaluation in large randomised studies or its use in clinical practice.

The results of the present phase I study, in which a purer and easier to produce semisynthetic form of HHT is given by s.c. route, prove that the regimen is feasible in patients with advanced/refractory acute leukaemia, with an acceptable toxicity profile.

Our pharmacokinetic data indicate low interpatient variability after s.c. administration of ssHHT. The volume of distribution, total plasma clearance and terminal elimination half-life of ssHHT are very close to pharmacokinetic data published with continuous intravenous administration of ssHHT. As an illustration, Savaraj *et al* (1986) explored the pharmacokinetic of continuous infusion of HHT at doses of 3–4 mg m^−2^ day^−1^. *V*_*d*_*β*/*F*, CL/*F* and *T*_1/2_ were 2.4±0.4 l kg^−1^, 12.4±1.9 l h^−1^ and 9.3±1.4 h, respectively. Semisynthetic homoharringtonine inhibits protein synthesis in a dose- and time-dependent manner. Peak plasma of ssHHT at 3 and 5 mg m^−2^ day^−1^ levels were above the concentrations required *in vitro* to inhibit 50% of the growth of human leukaemic HL-60 cells (Zhou *et al*, 1995).

The design of the trial used the CRM, a Bayesian sequential design, to determine the MTD. This method allowed us a precise estimation of the MTD, with an estimated toxicity probability at the 5 mg m^−2^ dosage of 36.1% (95% credibility interval: 15.8–58.6%), 12 patients being treated at this dose. This estimation compares favourably with classical estimation of the MTD in phase I trial using, for example, the standard 3+3 method in which a maximum of six patients are treated at the MTD.

This trial demonstrates that, in patients with advanced AML, the MTD of ssHHT is 5 mg m^−2^ day^−1^ during 9 days by s.c. route. This regimen is primarily associated with a haematological toxicity, with a prolonged myelosuppression and, in several cases, febrile neutropenia with life-threatening infection in two cases.

Antileukaemic activity was not the primary end point of this study, and patient population was heavily pretreated with few young patients or patients having been treated by allogeneic stem cell transplantation. Nevertheless, three out of 18 (17%) patients benefited from the treatment, experiencing either CR or return to a chronic phase of a CMML. Taken together with the toxicity profile of the drug, this suggests that ssHHT should be tested early in the course of the disease.

Subcutaneous dose schedule of ssHHT may provide easier administration for patients and will permit the exploration of novel dosing schedule in chronic disease such as MDS or AML in remission as maintenance therapy (Buchner *et al*, 2001). Moreover, consideration should be given to re-exploring the potential value of ssHHT in other haematological malignancies such as CML (O'Brien *et al*, 2003; Marin *et al*, 2005), specially in cases of bcr-abl mutation T315I, refractory to several tyrosine kinase inhibitors. Furthermore, manipulation in the side chain of ssHHT may provide second-generation analogues that may have a broader or different spectrum of activity.

In conclusion, the recommended dose of ssHHT by s.c. route is 5 mg m^−2^ day^−1^ for 9 days. Semisynthetic homoharringtonine is well tolerated and has a favourable pharmacokinetic profile with low interpatient variability. It merits further evaluation in phase II studies. A clinical development plan aiming for registration of ssHHT is currently ongoing, sponsored by ChemGenex Phamraceutical Ltd (CA, USA) and Stragen Pharma (Geneva, Switzerland).

## Figures and Tables

**Table 1:** Updated DLT probabilities of the five tested dose levels, computed after each newly included cohort of three patients per dose level through the use of Bayes theorem

			**HHT-loading dose (mg m^−2^ day^−1^)**				
			**Initial guesses of toxicity probability**				
			**0.5**	**1**	**3**	**5**	**6**
			**Updated estimated probability of DLT**				
**Cohort**	**Administrated dose (mg m^−2^ day^−1^)**	**Clinical response**	**0.5**	**0.1**	**0.15**	**0.33**	**0.50**
1	0.5	NT NT NT	0.001	0.003	0.006	0.035	**0.11**
2	3	NT NT T	0.07	0.14	0.19	**0.39**	0.55
3	5	NT NT T	0.07	0.13	0.19	**0.38**	0.54
4	5	NT NT NT	0.03	0.07	0.11	**0.27**	0.45
5	5	NT T NT	0.04	0.08	0.12	**0.29**	0.46
6	5	T NT T	0.06	0.12	0.17	**0.36**	0.53

DLT=dose-limiting toxicity; HHT=homoharringtonine; NT=no toxicity; T=DLT.

In bold: the dose level closest to the toxicity target (33%).

**Table 2a:** Summary of clinical toxicities during the trial

	**No of toxicities (grade 1/grade 2/grade 3/grade 4) at dose levels of**		
	**0.5 mg m^−2^ (*n*=three patients)**	**3 mg m^−2^ (*n*=three patients)**	**5 mg m^−2^ (*n*=12 patients)**
Cardiovascular	—	—	5/2/1/0
Cutaneous	2/0/0/0	1/0/0/0	6/1/2/0
Digestive	1/2/0/0	2/1/0/0	2/2/1/0
Endocrinian	—	0/0/1/0	2/4/0/0
Neurological	—	—	1/2/0/0
Infectious	1/1/0/0	0/0/1/0	1/1/0/1
Muscular	—	—	2/0/0/0
Pain	1/0/0/0	1/0/0/0	4/1/1/0
			
Total	5/3/0/0	4/1/2/0	23/13/5/1

**Table 2b:** Detailed grade 3 and grade 4 clinical toxicities per patient during the trial

**Patient no.**	**Dose (mg m^−2^)**	**Type of toxicity**	**Grade of toxicity**	**Details**	**DLT**
UPN 7	3	Endocrinian	3	Diabetes	Yes
		Infectious	3	Candidiasis septicaemia	No
UPN 10	5	Cardiovascular	3	Anasarque	Yes
		Digestive	3	Haematemesis	Yes
UPN 12	5	Infectious	3	Pneumonia	No
UPN 15	5	Infectious	4	Aspergillosis	Yes
UPN 17	5	Cutaneous	3	Rash	Yes
UPN 19	5	Pain	3	Scalp	Yes

DLT=dose-limiting toxicity.

**Table 3:** *In vivo* antileukaemic effect of ssHHT

**Patient no.**	**Dose (mg m^−2^)**	**Blood blast clearance**	**Bone marrow D10**	**Blood/bm at the end of cytopaenia**	**Result of treatment**
1	0.5	D8	94% blasts	Leukaemic	Blood blast clearance
2	0.5	No clearance	Not done	Leukaemic	No effect
3	0.5	Not evaluable	Not done	Leukaemic	No effect
4	3	D7	74% blasts	Leukaemic	Blood blast clearance
6	3	Not evaluable	63% blasts	Leukaemic	No effect
7	3	D4	Clearance	Death	Blood and bm clearance
8	5	D6	70% blasts	Leukaemic	Blood blast clearance
9	5	D11	Clearance	15% in bm	Blood and bm clearance
10	5	D7	Not done	Death	Blood blast clearance
11	5	Not evaluable	76% blasts	18% in bm	Reduction of bone marrow blasts
12	5	Not evaluable	25% blasts	Leukaemic	No effect
13	5	D3	Clearance	Aplastic at D36	Blood and bm clearance
14	5	D5	Clearance	4% in bm	CR (4 months)
15	5	D4	Not done	4% in bm	CR (3 months)
16	5	D6	41% blasts	42% in bm	Blood blast clearance
17	5	Not evaluable	12% blasts	5.5% in bm	Blood and bm clearance
18	5	D7	Clearance	7.5% in bm	Return to CMML (6 months)
19	5	D6	Not done	Aplastic at D38	Blood and bm clearance

CMML, chronic myelomonocytic leukaemia; CR, complete remission.

**Table 4:** HHT Pharmacokinetic parameters

**Dose**			***C*_max_ day 5 (ng ml^−1^)**		***T*_max_ day 5 (h)**		***C*_min_ day 5 (ng ml^−1^)**		***C*_min_ day 9 (ng ml^−1^)**		**AUC day 9 (ng h ml^−1^)**		***V_d_β*/*F* (l kg^−1^)**		**CL/*F* (l h^−1^)**		***T*_1/2_ (h)**	
**mg m^−2^ day^−1^**	**mg**	**No. Pts**	**Mean**	**s.d.**	**Mean**	**s.d.**	**Mean**	**s.d.**	**Mean**	**s.d.**	**Mean**	**s.d.**	**Mean**	**s.d.**	**Mean**	**s.d.**	**Mean**	**s.d.**
0.5	0.9–1.0	3	10.5	8.8	1.0	0.12	3.4	0.9			169	38	1.52	0.39	5.9	1.60	13.4	3.8
3	4.8–5.4	2	78.0	18.2	0.6	0.53	27.4	27.9	14.7	8.3	441	291	2.54	0.75	14.2	8.77	8.9	3.7
5	7.0–10.0	12	96.1	20.3	0.6	0.43	31.3	16.7	25.6	20.3	909	550	2.02	1.43	11.6	10.4	10.8	3.2
																		
Grand mean																	11.01	3.4*

* *P-value for Kruskall–Wallis test (*P*=0.52).
